# Mesoporous Silica Nanoparticles Functionalized with Bisphenol A for Dispersive Solid-Phase Extraction of 3-Chloroaniline from Water Matrices: Material Synthesis and Sorption Optimization

**DOI:** 10.3390/nano15231751

**Published:** 2025-11-22

**Authors:** Sultan K. Alharbi, Bandar R. Alsehli, Awadh O. AlSuhaimi, Khaled A. Thumayri, Khaled M. AlMohaimadi, Yassin T. H. Mehdar, Manal A. Almalki, Belal H. M. Hussein

**Affiliations:** 1 Chemistry Department, Faculty of Science, Taibah University, Madinah Al-Munawaroh 41321, Saudi Arabia; sbdrani@taibahu.edu.sa (S.K.A.); bshle@taibahu.edu.sa (B.R.A.); kthumairi@taibahu.edu.sa (K.A.T.); belalhussein102@yahoo.com (B.H.M.H.); 2 Ministry of Education, Director General of Education, Madinah Al-Munawaroh 42314, Saudi Arabia; khaled-mohaimadi@hotmail.com; 3 Chemistry Department, Faculty of Science, Suez Canal University, Ismailia 41522, Egypt

**Keywords:** mesoporous silica nanoparticles, bisphenol-A functionalization, 3-chloroaniline, dispersive solid-phase extraction, water treatment, environmental analysis

## Abstract

Aromatic amines such as 3-chloroaniline (3-CA) are toxic, persistent, and environmentally relevant water contaminants. Their reliable determination in aqueous systems has therefore become increasingly important. The monitoring of trace levels of these pollutants in complex water matrices typically necessitates a preconcentration step, most achieved via solid-phase extraction (SPE). However, conventional SPE sorbents often suffer from limited surface reactivity and slow adsorption kinetics, which compromise their performance at ultra-low concentrations. In contrast, nanomaterials offer a promising upgrade due to their high surface area, tunable chemistry, and rapid mass transfer behavior. In this work, mesoporous silica nanoparticles (MSNs) were synthesized via a green sol–gel route from sodium silicate precursor using polyethylene glycol template and then chemically functionalized with bisphenol A (BPA) to produce BPA-MSNs with π-rich and hydrogen-bonding active sites. Characterization using XRD, BET, FTIR, SEM/EDX, and TGA confirmed the successful synthesis and surface modification of the nanosorbent. BPA-MSNs achieved a maximum adsorption capacity of 30.2 mg/g toward 3-CA, fitting Langmuir and Jovanovic isotherm models. Kinetic analysis followed a pseudo-first-order model, indicating physisorption enhanced by π–π stacking and hydrogen bonding. The optimized dispersive SPE (D-SPE) method allowed a low detection limit (LOD = 0.016 mg/L), recovery of 73–85%, and precision below 5.3% RSD in tap, bottled, synthetic municipal wastewater and groundwater samples. The sorbent retained >90% efficiency over five reuse cycles, demonstrating strong potential as a reusable nanosorbent for preconcentration and remediation of aromatic amines in and treatment water analysis.

## 1. Introduction

Aromatic amine derivatives (AADs), such as 3-chloroaniline (3-CA), o-toluidine, and p-chloroaniline, are significant environmental pollutants due to their widespread use as intermediate substances in the production of many products including dyes, rubber chemicals, insecticides, pharmaceuticals, and other industrial substances [1,2,3]. These compounds are released into the environment through industrial effluents, improper disposal, and as degradation byproducts, notably from herbicides like chlorpropham (CIPC), which breaks down into chloroaniline derivatives [4]. Their resistance to biodegradation leads to accumulation in aquatic systems, soils, and sediments, thereby posing potential toxicity risks to aquatic organisms and human health [5]. Notably, 3-CA is highly toxic to aquatic life, bioaccumulates in food chains, and is classified as a probable carcinogen [6,7]. Its persistence, bioaccumulation potential, and frequent detection across diverse environmental matrices make it a critical concern among the AADs [8,9,10].

The widespread of these pollutants in environmental systems highlights the critical need for robust monitoring strategies to address contamination and advance environmental sustainability [11,12]. However, their analysis remains challenging due to their low concentration and the complexity of environmental samples. Thus, appropriate sample treatment procedures able to preconcentrate and selectively isolate these analytes from complex interferences are usually required. Various methods have been employed for these purposes, including solid-phase extraction (SPE) [13], solid-phase microextraction (SPME) [14], dispersive liquid–liquid microextraction (DLLME) [15], and dispersive solid-phase extraction (D-SPE) [16]. Of these methods, adsorbent-supported extraction techniques using adsorbents such as activated carbon [17], mesoporous silica, clay [18,19], and graphene oxide [20] are extremely effective in separating and pre-concentrating chloroanilines from a wide variety of matrices [21,22]. Many of these materials have high surface areas and structurally tunable pore structures, which enable selective adsorption and easy subsequent elution for analysis or recovery.

Over the past years, nanostructured materials have gained increased applications as effective solid substrates for SPE, especially in dispersive micro solid-phase extraction (D-µ-SPE) mode. Nowadays, the D-µ-SPE method, in which nanosorbents are merely dispersed in sample solutions for rapid and effective extraction of the analytes, is very popular in environmental and analytical chemistry for the extraction of organic pollutants from complex samples [23,24]. The widespread adoption of D-µ-SPE can be attributed to the exceptional properties of nanomaterials, such as their high surface area, uniform particle size distribution, and customizable surface chemistry, which collectively enhance extraction efficiency and selectivity [24].

Mesoporous silica nanoparticles (MSNs) have emerged as highly versatile nanomaterials owing to their ordered mesostructure, tunable pore size, and exceptionally high surface area (>1000 m^2^ g^−1^). These features, coupled with remarkable thermal and chemical stability under varying pH and temperature conditions, render MSNs excellent nanosorbents for the extraction/removal of contaminants from diverse environmental matrices [25,26,27]. Their large pore volume and accessible surface provide numerous adsorption sites, while surface functionalization with amine, thiol, or phenyl groups enhances selectivity toward specific analytes. Accordingly, MSNs have been successfully applied to the removal of polycyclic aromatic hydrocarbons, pharmaceuticals, and endocrine-disrupting compounds from aqueous systems [28,29,30].

Conventionally, MSN has been synthesized via sol–gel chemistry from alkoxysilane precursors like tetraethyl orthosilicate (TEOS) in presence of structure-directing agents (SDAs) to define pore architecture [31]. Despite offering precise morphological control, alkoxysilanes are hampered by high cost and limited scalability. On the other hand, alkali silicate solutions provide a sustainable cost-effective alternative with higher silica content, faster condensation kinetics, and superior scalability [32,33,34]. The MSNs obtained from alkali silicate exhibit excellent mechanical stability and structural precision, comparable to alkoxysilane-derived MSNs.

The selection of SDA critically governs pore ordering, size distribution, and surface chemistry. While cationic surfactants like CTAB and CTAC are widely used templates, their toxicity and difficult removal present significant drawbacks [35]. Therefore, eco-friendly alternatives like polyethylene glycol (PEG) have attracted more attention as non-toxic biodegradable templating, while simultaneously acting as pore-forming and stabilizing agents. PEG maintains ordered mesostructures and high surface areas while enabling environmentally benign synthesis routes.

The sol–gel process provides a versatile platform for the synthesis of mesoporous silica nanoparticles (MSNs) under a range of experimental conditions. Among these methodologies, hydrothermal-assisted sol–gel synthesis has emerged as the most efficacious route for fabricating highly ordered MSNs with well-defined mesostructures. This technique leverages elevated temperature and pressure to accelerate the condensation kinetics of silica species while facilitating the thermodynamic reorganization of the surfactant-silica composite into crystalline mesophases with minimal structural defects [36,37]. Furthermore, the strategic combination of alkali silicate precursors with green SDAs, such as PEG, enables a cost-effective and environmentally sustainable production pathway for these nanomaterials.

This study presents a strategic advancement in the design of functional nanomaterials for the targeted sequestration of 3-CA from water. Building upon our previous work [33], we have modified the established hydrothermal-assisted sol–gel synthesis by employing PEG as the sole SDA. This targeted modification eliminates the need for cytotoxic surfactants, streamlining the template removal process and enhancing the sustainability of the MSN platform. The principal innovation, however, lies in the subsequent pioneering functionalization of these MSNs with bisphenol A (BPA) via a novel multi-step post-synthetic route, a strategy previously unreported in the literature. This deliberate surface engineering creates a π-rich interface with specific hydrogen-bonding sites, engineered for superior affinity and selectivity towards aromatic amine contaminants. The efficacy of the resulting BPA-MSNs was systematically investigated through sorption optimization, kinetic, and isotherm analyses. The practical application was ultimately demonstrated by the successful implementation of the material as an efficient sorbent in D-SPE for the effective recovery of 3-CA from various aqueous matrices, thereby establishing a new design paradigm for selective nano adsorbents.

## 2. Materials and Methods

### 2.1. Chemical Reagents

All reagents employed in the synthesis of silica nanoparticles, the immobilization of bisphenol A, and the adsorption process were of analytical grade. Sodium silicate solution (Na_2_O·3SiO_2_; Na_2_O, 8%; SiO_2_, 27%) was donated by Adwan Chemical Industries Co., Ltd. (Riyadh, Saudi Arabia). Poly(ethylene glycol) (M.W. 20,000) and bisphenol A were obtained from Acros Organics (Geel, Belgium). Trimethoxyphenylsilane and anhydrous toluene were acquired from Sigma-Aldrich (St. Louis, MO, USA). Hydrochloric acid and sodium hydroxide were sourced from Simnt (Riyadh, Saudi Arabia). Ammonium acetate, ethanol, and sodium dithionite (Na_2_S_2_O_4_) were purchased from PanReac (Barcelona, Spain), while nitric acid and ammonium hydroxide solution were obtained from Loba Chemie (Mumbai, India).

### 2.2. Instrumentations

X-ray diffraction (XRD) patterns were obtained using a Rigaku Multiflex diffractometer (Rigaku Corporation, Tokyo, Japan) equipped with monochromated high-intensity Cu Kα radiation (λ = 1.54 Å). Scanning was performed under ambient conditions over the 2θ range of 0.7–5° at a rate of 0.1°/min (20 kV, 10 mA). N_2_ adsorption–desorption isotherms and pore characterization were obtained using a Micromeritics ASAP 2020 apparatus (Micromeritics Instrument Corporation, Norcross, GA, USA) at liquid nitrogen temperature. The specific surface areas (SBET) of the samples were determined from the linear segment of the BET plot, while the Barrett–Joyner–Halenda (BJH) method was employed to determine the pore size distributions. Field emission scanning electron microscopy (FESEM) images were acquired using a Zeiss EM10C-100 kV instrument (Carl Zeiss, Oberkochen, Germany), and the average particle size was determined using ImageJ software (version 1.54g) (National Institutes of Health, Bethesda, MD, USA). Prior to measurement, the samples were dispersed onto a steel plate surface and coated with Pt metal. Fourier transform infrared (FTIR) spectra were obtained using a Thermo Fisher Scientific Inc. spectrometer (Waltham, MA, USA), and thermogravimetric analysis (TGA) was performed using a NEXTA STA200 analyzer (Hitachi High-Tech Corporation, Tokyo, Japan) to evaluate the weight loss as a function of temperature. Concentrations of 3-chloroaniline were measured using a Shimadzu UV-1800 spectrophotometer (Shimadzu Corporation, Kyoto, Japan).

### 2.3. Synthesis of MSNs

MSNs were synthesized from sodium silicate solution using the previously reported hydrothermal-assisted sol–gel method [33], but without using CTAC as a structure-directing agent. Briefly, polyethylene glycol (PEG, 1 mM) was dissolved in 50 mL of water containing 3.0 mL NH_4_OH at 40–50 °C. Separately, a commercial sodium silicate solution (SSS) was diluted to 0.2 M and adjusted to pH 9 with 1 M HNO_3_; then, 50 mL of this solution was passed through a cation-exchange resin to remove sodium and other cations. This purified SSS (50 mL) was then added dropwise to the PEG/NH_4_OH solution under stirring (300 rpm, 1 h). The pH was adjusted to 11.5 with ammonia, bringing the total reaction volume to 150 mL. The mixture was transferred to an autoclave and heated at 120 °C for 12 h. The resulting white precipitate was collected by centrifugation (4000 rpm, 10 min), washed repeatedly with deionized water and ethanol, and calcined at 550 °C to remove organic templates.

### 2.4. Functionalization of MSNs with Bisphenol-A Moiety

The synthesis of bisphenol A-functionalized mesoporous silica nanoparticles (BPA-MSNs) proceeded via sequential surface modifications starting from pristine MSNs. Initially, 1 g of plain MSNs was dispersed in 25 mL anhydrous toluene under stirring, and 0.5 mL 3-trimethoxyphenylsilane (TMPS) was added dropwise. The mixture was refluxed for 8 h, and then, the produced phenyl-functionalized silica (PhO-MSNs) was centrifuged (4000 rpm, 10 min.) and repeatedly washed with ethanol and oven dried at 70 °C. The dried PhO-MSNs was subjected to nitration using a mixed acid system comprising concentrated nitric acid and sulfuric acid (10:20 mL, *v*/*v* 1:2). The reaction was conducted at 60 °C for 1 h in a water bath, facilitating the electrophilic substitution of nitro groups onto the aromatic rings. The resulting solid was isolated by filtration and thoroughly washed with distilled water to remove residual acids. The surface nitro groups were reduced to primary amines using 50 mL of 5% (*w*/*v*) aqueous sodium dithionite in a sealed flask at 45 °C for 24 h. The resulting suspension was cooled to 0–5 °C (±0.2 °C), acidified with 3 mL of 2 M HCl for 30 min, and diazotized by adding 2.0 mL of cold aqueous NaNO_2_ with stirring for 10 min. A 50 mL aliquot of 2% (*w*/*v*) bisphenol A in ethanol was then added dropwise, and the coupling reaction proceeded for 4 h. The final BPA-MSNs were isolated by centrifugation (4000 rpm, 10 min), vacuum-dried, and stored for further use.

### 2.5. Adsorption and D-SPE Procedure

Adsorption studies were conducted using batch adsorption experiments by dispersing 100 mg of BPA-MSNs into 100 mL of 3-CA solution, with the equilibrium concentration measured spectrophotometrically at 235 nm. The effects of pH (2–9) were examined to determine the optimal adsorption conditions, which were subsequently applied for the D-SPE procedure. The adsorption behavior was interpreted using six isotherm models (Langmuir, Freundlich, Temkin, Sips, Jossens, and Jovanovic) and four kinetic models (pseudo-first-order, pseudo-second-order, Elovich, and intraparticle diffusion) to elucidate the mechanism and rate of 3-CA uptake by BPA-MSNs. For D-SPE experiments, 50 mg of sorbent was dispersed into 100 mL of a water sample, which was then centrifuged and eluted with 10 mL of ethanol. The regeneration of the sorbent was attained by washing with HNO_3_ (1 M) and water and then vacuum-drying before reuse.

## 3. Results

### 3.1. Synthesis and Characterization of Sorbent

MSNs with spherical to cashew-like morphologies were synthesized via the modified hydrothermal-assisted sol–gel methodology developed in this work. It employs sodium silicate (Na_2_SiO_3_) as the inorganic precursor and PEG as an environmentally benign nonionic SDA [38]. The reaction was conducted under strongly alkaline conditions (pH ≈ 11.5), wherein silicate species predominantly exist as deprotonated monomeric and low-molecular-weight oligomeric anions (e.g., [SiO(OH)_3_]^−^ and [SiO_2_(OH)_2_]^2−^), thereby facilitating kinetically controlled hydrolysis and condensation processes.

The proposed mechanism involves electrostatic and hydrogen-bonding interactions between anionic silicate species and protonated or hydrated PEG chains, leading to the formation of hybrid organic–inorganic composite micelles. These supramolecular assemblies undergo cooperative self-organization into an ordered mesophase, with PEG imparting steric stabilization and templating the pore architecture [39,40]. Subsequent hydrothermal aging enhances silica polycondensation and framework cross-linking, followed by calcination to selectively remove the organic template, yielding a robust high-surface-area mesoporous silica network with well-defined porosity [33].

The synthesized MSNs underwent a multi-step post-synthesis chemical modification to tailor their surface properties. This process began with silylation using TMPS in anhydrous toluene to append aryl groups, followed by nitration (H_2_SO_4_/HNO_3_) and reduction with sodium dithionite (Na_2_S_2_O_4_) to yield aniline-terminated surfaces. Subsequent in situ diazotization (NaNO_2_/HCl, 0–5 °C) and immediate azo-coupling with BPA produced BPA-MSNs through covalent aryl-azo linkages.

**Figure 1:**
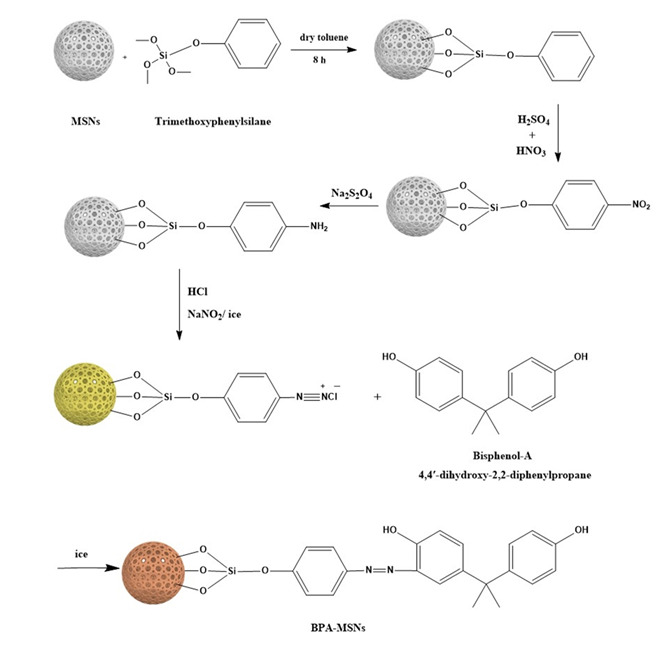
Reaction scheme for the covalent attachment of bisphenol-A onto MSNs, showing the associated color changes from white (pristine MSNs) to pale yellow (aniline-MSNs) and finally to intense orange (BPA-MSNs) upon successful azo-coupling.

The success of this post-synthesis chemical modification was qualitatively evident from the distinct color transitions observed after key synthetic steps. The formation of the aniline groups imparted a pale-yellow color to the particles, while the subsequent azo-coupling reaction with BPA resulted in an intense orange hue, characteristic of the formation of the extended chromophoric azo bond (–N=N–) (see Figure 1).

The success of MSNs and their functionalization were confirmed by a suite of characterization techniques, including XRD (crystallinity), SEM-EDX (morphology, elemental mapping), N_2_ physisorption (textural properties), FTIR (surface chemistry), and TGA (organic loading and thermal stability) [41,42,43]. This diazonium-mediated grafting ensures high-density hydrolytically stable organic–inorganic interfaces [42], with BPA’s phenolic –OH and aromatic domains enhancing π–π and hydrogen-bonding interactions for targeted adsorption of aromatic amines such 3-CA.

**Figure 2:**
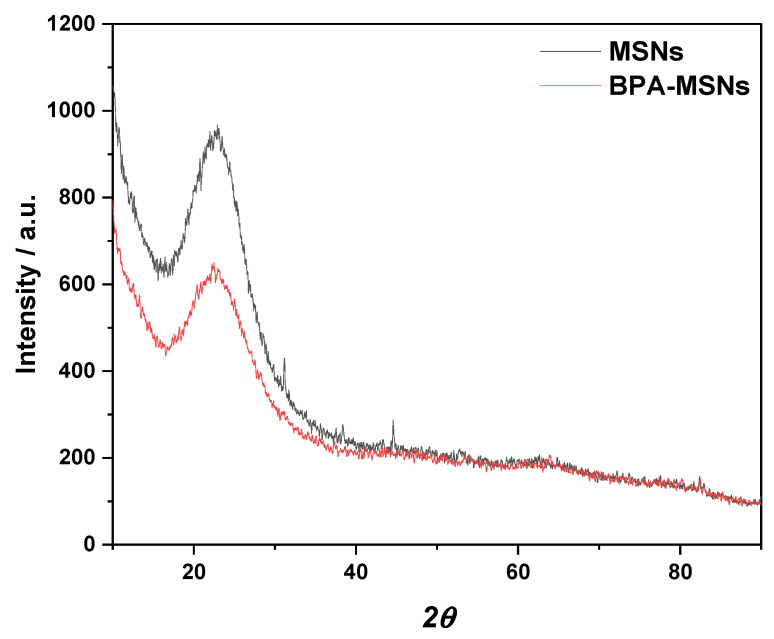
XRD patterns of calcined MSNs and BPA-MSNs, showing the characteristic broad peak of amorphous silica and its attenuation after surface functionalization.

Structural analysis by XRD (Figure 2) confirmed the amorphous nature of the silica matrix, with calcined MSNs exhibiting a characteristic broad peak at 2θ ≈ 22.6°, corresponding to the amorphous silica framework [32]. After functionalization, BPA-MSNs maintained this characteristic feature but exhibited a measurable reduction in peak intensity compared to the calcined MSNs. This attenuation reflects a minor introduction of structural disorder upon the incorporation of organic moieties, consistent with observations in other modified mesoporous silicas, where surface grafting disrupts long-range electron density uniformity without compromising the structural integrity of the framework [31,44].

**Figure 3:**
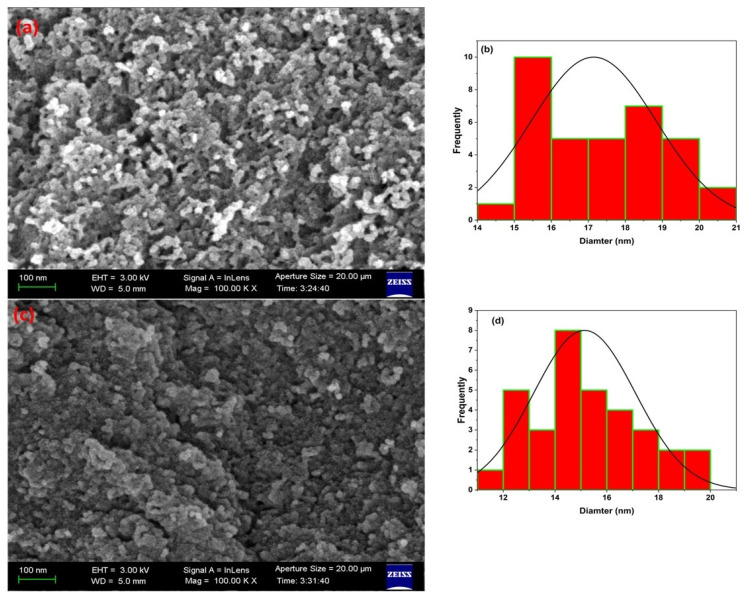
SEM images of (**a**) MSNs exhibiting spherical to cashew-like morphologies and (**c**) BPA-MSNs after surface functionalization. The corresponding particle size distribution histograms are shown in (**b**,**d**), respectively, illustrating narrow diameter ranges consistent with the SEM observations.

The SEM images (Figure 3a,c) reveal that the synthesized MSNs display morphologies spanning quasi-spherical to cashew-shaped profiles, with a narrow particle-size distribution of 12–21 nm, as corroborated by statistical analysis (Figure 3b,d). These dimensional and morphological characteristics align closely with prior reports on PEG-templated silica nanoparticles [36,37]. After surface functionalization with bisphenol-A (BPA), the mean particle diameter exhibits a modest reduction from ≈17 nm (calcined pristine MSNs) to ≈15 nm (BPA-MSNs), indicative of limited framework contraction during the multi-step covalent grafting sequence. Critically, the overall particle morphology and monodisperse nanoscale size distribution remain essentially unaltered post-modification, demonstrating that the BPA immobilization protocol preserves structural integrity without inducing aggregation or significant geometric distortion.

**Figure 4:**
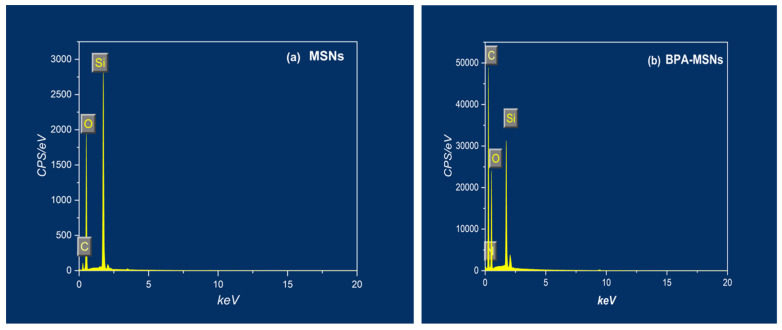
EDX spectra of (**a**) MSNs and (**b**) BPA-MSNs. Pristine MSNs show characteristic signals for Si and O, whereas BPA-MSNs exhibit an increased carbon signal consistent with successful BPA functionalization of the silica surface.

The EDX spectra (Figure 4, Table 1) provide definitive evidence of the successful surface functionalization. The spectrum of the BPA-MSNs exhibits distinct peaks for carbon and nitrogen, which are absent in the pristine MSNs, consistent with the literature on amino- and phenyl-modified silica [27,28]. Silicon and oxygen are the dominant elements, with a measured Si:O atomic ratio of approximately 1:2, confirming the preserved SiO_2_ framework wherein the silane-based functionalities are primarily surface-localized. Quantitative analysis determined the atomic percentages of oxygen and silicon to be 68.8% and 31.2%, respectively. Critically, the BPA-MSNs spectrum revealed significant atomic percentages of 60.31% for carbon and 4.25% for nitrogen, providing direct quantitative confirmation of the successful grafting of the azo compound onto the mesoporous silica surface.

**Figure 5:**
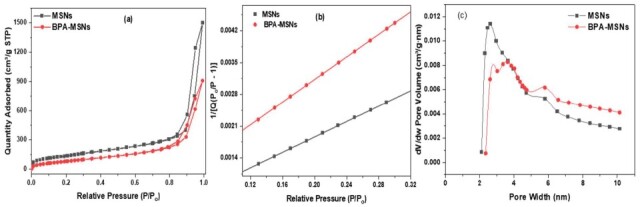
N_2_ adsorption–desorption isotherms of MSNs and BPA-MSNs (**a**), with corresponding BET linear plots (**b**). The BJH pore size distribution curves (**c**) demonstrate the shift in pore characteristics following BPA functionalization.

Nitrogen adsorption–desorption isotherms (Figure 5) displayed type-IV hysteresis loops, which confirmed the mesoporosity of the material [30]. The BET surface area decreased from 503 to 334 m^2^/g and the pore volume from 1.9 to 1.4 cm^3^/g because of the chemical functionalization (Table 2). This is attributed to the partial blocking of mesopores by the grafted BPA moieties, as observed in other functionalized silicas [30,31]. The slight increase in average pore size from 2.6 to 3.6 nm also suggests restructuring of the pore environment due to grafting [44].

The FTIR spectrum of pristine MSNs exhibited a broad band at 3500–3000 cm^−1^, corresponding to O–H stretching vibrations of surface silanol (Si-OH) groups and adsorbed water. The peak at 1635 cm^−1^ was assigned to H–O–H bending of molecular water, while intense bands at 1057 and 798 cm^−1^ were attributed to asymmetric and symmetric Si–O–Si stretching vibrations, respectively. Additional peaks at 958 cm^−1^ (Si-OH bending) and 453 cm^−1^ (Si-O-Si rocking) confirmed the silicate framework [45].

**Figure 6:**
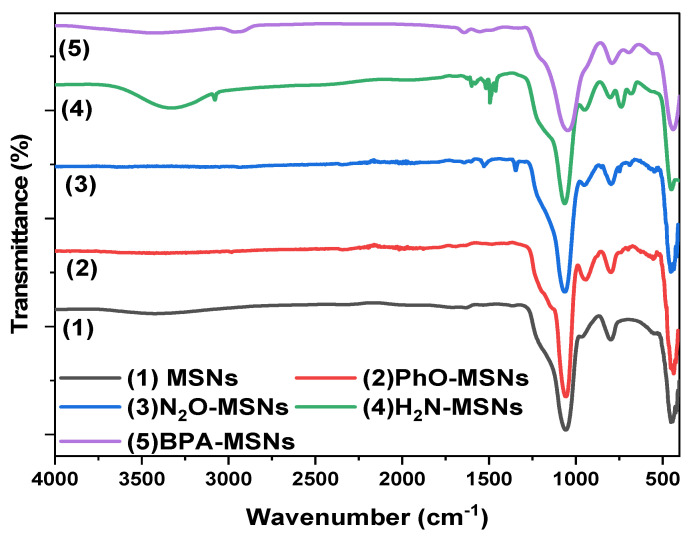
FTIR spectra of the synthesized materials: (1) calcined MSNs, (2) TMPS-silanized MSNs, (3) nitrated intermediate, (4) reduced amine-functionalized product, and (5) final BPA-MSNs. The sequential spectral changes confirm each modification step during surface functionalization.

After silanization with TMPS, a new absorption band appeared at 3040 cm^−1^, corresponding to aromatic =C-H stretching, verifying the successful grafting of phenyl groups. Subsequent nitration introduced a characteristic band at 1525 cm^−1^ from asymmetric N–O stretching of the -NO_2_ group. Upon reduction, this band disappeared and was replaced by a broad absorption near 3325 cm^−1^, attributed to N-H stretching vibrations of primary amines, confirming the conversion of -NO_2_ to -NH_2_ functionalities [46,47]. In the FTIR spectrum of BPA-MSNs, the silanol band intensity markedly decreased, while new aliphatic C-H and phenolic -OH stretching bands emerged, confirming bisphenol A immobilization. The sequential spectral changes are illustrated in Figure 6.

**Figure 7:**
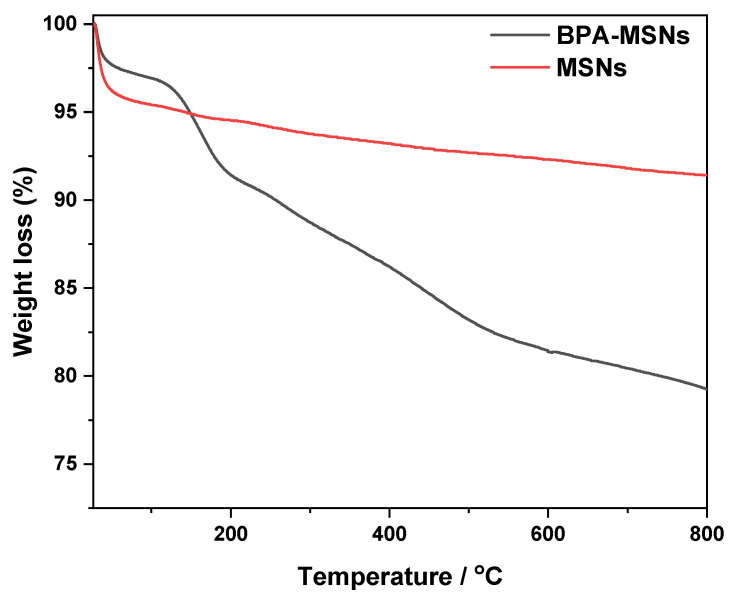
Thermogravimetric analysis of samples: as-made MSNs (red line) and after functionalization with BPA (black line).

TGA (Figure 7) further validated the functionalization. Calcined MSNs showed negligible weight loss above 180 °C, reflecting the removal of PEG. In contrast, BPA-MSNs exhibited significant weight losses between 150 and 650 °C, attributed to the decomposition of organic fragments [48,49]. The higher organic loading corroborates the FTIR and BET results, confirming successful grafting.

### 3.2. Adsorption Performance and the Influence of Solution pH

The adsorption efficacy of the synthesized BPA-MSNs for 3-CA was first quantified using the equilibrium adsorption capacity (q_e_) and removal efficiency (η%), as defined in Equations (1) and (2).

(1) $$q_{e}=\frac{\left(C_{i}-C_{e}\right)V}{m}$$

(2) $$\%\eta =\left[\frac{C_{i}-C_{e}}{C_{i}}\right]\times 100$$

These initial measurements revealed a strong dependence on the solution pH, a key factor governing the electrostatic interactions between the adsorbent and adsorbate.

**Figure 8:**
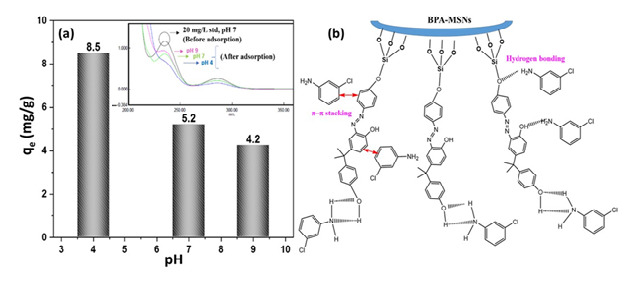
(**a**) Effect of initial pH on the adsorption capacity of 3-CA onto BPA-MSNs. (Initial concentration = 20 mg L^−1^, contact time = 60 min, adsorbent dosage = 0.1 g, temperature = 25.5 °C). (**b**) Proposed interaction mechanism between BPA-MSNs and 3-chloroaniline.

The maximum adsorption capacity was observed at pH 4 (Figure 8a). This optimum can be attributed to the speciation of 3-CA (pK_a_ ≈ 4.0) and the surface charge of the MSNs. Under acidic conditions (pH ≤ 4), 3-CA is predominantly protonated, acquiring a positive charge, while the silanol groups (Si-OH) on the MSN surface are neutral or partially deprotonated, resulting in a negative surface charge. This creates a favorable condition for strong electrostatic attraction, which serves as the primary driving force for adsorption [50]. As the pH increases beyond 4, 3-CA becomes increasingly deprotonated and neutral, drastically reducing the electrostatic driving force and leading to the observed sharp decline in adsorption capacity.

In addition to electrostatics, the functionalization with BPA introduces secondary interaction sites. The aromatic rings of BPA facilitate π–π stacking with the 3-CA molecule, while the hydroxyl groups can engage in hydrogen bonding with the amine group of 3-CA (Figure 8b). These synergistic interactions—electrostatic attraction, π–π stacking, and hydrogen bonding—collectively underpin the high affinity and selectivity of BPA-MSNs for chloroaniline compounds [51]. The mesoporous structure ensures a high surface area and facilitates the rapid diffusion of 3-CA molecules to these binding sites.

### 3.3. Equilibrium Isotherm Analysis and Surface Interaction Mechanisms

The equilibrium adsorption data for 3-CA onto the biphasic BPA-MSNs were analyzed using six nonlinear isotherm models (Langmuir [52], Freundlich [53], Temkin [54], Sips [55], Jossens [56], and Jovanovic [57]) to elucidate the underlying surface interaction mechanisms. The selection of these models aimed to discriminate between key adsorption hypotheses: monolayer versus multilayer formation, homogeneous versus heterogeneous surface energy, and the role of adsorbate interactions.

**Figure 9:**
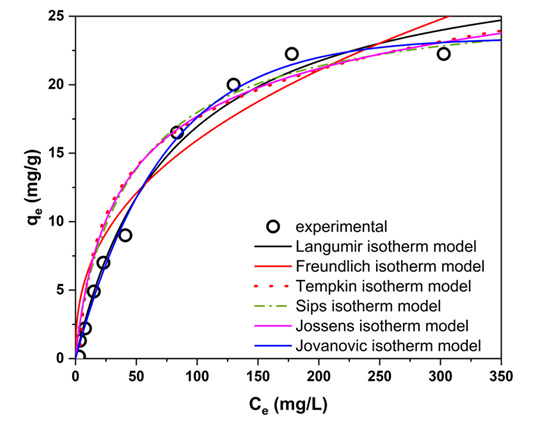
Nonlinear regression fits of experimental equilibrium data for 3-CA adsorption onto BPA-MSNs using six isotherm models. (Contact time = 60 min, adsorbent dosage = 0.1 g, pH = 4, temperature = 25.5 °C).

The fitting parameters and correlation coefficients are summarized in Table 3, and the corresponding curves are presented in Figure 9. The analysis of the obtained data reveals that the adsorption process is best described by monolayer coverage. The Jovanovic model provided the optimal fit (R^2^ = 0.99, RMSE = 0.72), closely followed by the Langmuir (R^2^ = 0.98, RMSE = 0.95) and Jossens (R^2^ = 0.98, RMSE = 0.85) models. The high performance of both the Langmuir and Jovanovic models indicates an adsorption system dominated by a monolayer mechanism on a surface with largely uniform site energies [58,59]. The superior fit of the Jovanovic model further suggests significant interaction strength, leading to a degree of desorption resistance.

The strong performance of the Jossens model acknowledges a minor degree of surface heterogeneity, likely intrinsic to the functionalized silica framework [57]. However, the distinctly poorer fits of the purely heterogeneous Freundlich and Temkin models confirm that heterogeneity is not the dominant characteristic. The hierarchy of model fits (Jovanovic > Jossens ≈ Langmuir > Sips > Freundlich > Temkin) conclusively describes a system of monolayer adsorption on a surface with predominantly uniform high-affinity sites. The favorable nature of the adsorption is indicated by the Freundlich exponent (n = 2.1 > 1). The near-identical performance of the Langmuir and Jovanovic models can be attributed to the minimal energetic heterogeneity of the BPA-MSNs surface, which renders the additional mechanical contact parameter in the Jovanovic equation negligible over the studied concentration range [58,59]. This suggests a well-defined and uniform porous structure. Therefore, the primary mechanism is favorable physisorption, driven by synergistic interactions including hydrogen bonding and van der Waals forces within the mesoporous channels [60].

### 3.4. Adsorption Kinetics and Rate-Limiting Steps

The adsorption kinetics were investigated to determine the rate-limiting step and elucidate the governing mechanism. Four established models were fitted to the experimental data using non-linear regression: the pseudo-first-order (PFO), pseudo-second-order (PSO), Elovich, and intraparticle diffusion (IPD) models. Application of multiple kinetic models is widely recommended in adsorption studies to rigorously discriminate between possible mechanisms without a priori assumptions [61,62,63,64,65].

The PFO model [61] was employed to test for physisorption governed by the availability of vacant surface sites. The PSO model [62] was included to evaluate potential chemisorption involving electron sharing or chemical bond formation. The Elovich equation [63] was applied to assess the influence of significant energetic heterogeneity on the adsorbent surface. Finally, the IPD model [64] was used to examine whether intraparticle pore diffusion constitutes the rate-limiting step.

**Figure 10:**
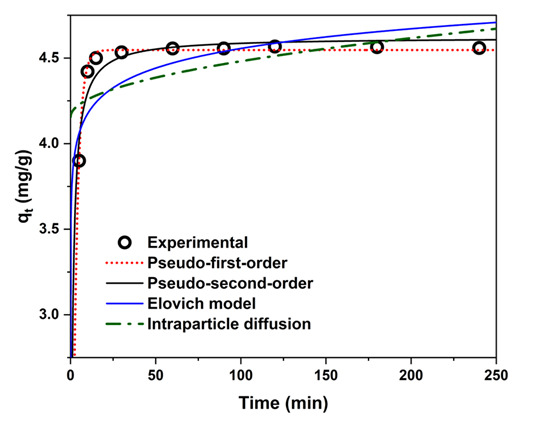
Kinetic models for the adsorption of 3-CA onto BPA-MSNs: pseudo-first-order, pseudo-second-order, Elovich, and intraparticle diffusion. (Initial concentration = 20 mg L^−1^, contact time = 60 min, adsorbent dosage = 0.1 g, pH = 4, temperature = 25.5 °C).

The fitted parameters and statistical metrics are presented in Table 4, and the corresponding regression curves are shown in Figure 10. Goodness-of-fit was assessed by the coefficient of determination (R^2^), root-mean-square error (RMSE), and the closeness of the calculated (q_e,cal_) and experimental (q_e,exp_) equilibrium adsorption capacities.

The PFO model provided the best fit (R^2^ = 0.999, RMSE = 0.022 mg g^−1^), with q_e,cal_ virtually identical to q_e,exp_ (Table 4). In contrast, the PSO model yielded a markedly poorer fit (R^2^ = 0.88), effectively ruling out chemisorption as the dominant mechanism. The Elovich and IPD models also exhibited low correlation coefficients and large deviations in predicted equilibrium capacity, indicating that neither surface energetic heterogeneity nor intraparticle mass-transfer resistance controls the adsorption rate. The negligible role of intraparticle diffusion further highlights the efficiency of the mesoporous network, which ensures rapid and unimpeded access to the adsorption sites.

These results provide compelling evidence that the adsorption process is predominantly physisorptive, with the rate controlled primarily by the concentration of unoccupied surface sites.

### 3.5. BPA-MSNs as D-SPE Sorbent for 3-CA: Calibration and Real-Sample Application

D-SPE using the synthesized BPA-MSNs nano adsorbent was optimized for the preconcentration of 3-CA from aqueous matrices, with quantification by UV–Vis spectrophotometry (λ_max_ = 240 nm). The method yielded a linear calibration range of 0.05–15.0 mg L^−1^ (A = 0.05929C − 0.000273, R^2^ = 0.9998, n = 7). The slope (m = 0.05929 L mg^−1^) indicates adequate sensitivity for trace analysis, while the near-zero intercept supports baseline reliability. Table 5 presents the analytical figures of merit for the BPA-MSNs–DSPE method for the extraction 3-CA. The limit of detection (LOD = 3 σ/m) was 0.016 mg L^−1^, and the limit of quantification (LOQ = 10 σ/m) was 0.05 mg L^−1^ (σ = standard deviation of blank, n = 10). The extraction of 100 mL of the sample with 100 mg BPA-MSNs, followed by elution in 10 mL ethanol, achieved a preconcentration factor (PF) of 9.54 close to the theoretical maximum of 10 with a recovery of 95.4 ± 2.1% (n = 3) (Table 5). This performance reflects efficient analyte–sorbent interaction, driven by molecular imprinting within a high-surface-area mesoporous framework (>500 m^2^ g^−1^).

UV–Vis detection, while simple and cost-effective, lacks selectivity in complex matrices containing UV-absorbing interferents (e.g., natural organic matter). To evaluate the matrix effects, river and lake water samples were spiked at 0.1–5.0 mg L^−1^, yielding recoveries of 92.7–106.4% (RSD ≤ 4.8%, n = 3) using standard addition. The analyte stability was confirmed over 24 h at 25 °C, with no significant change in absorbance or spectral shape.

The method’s applicability was validated, making use of recovery experiments in bottled, ground, tap, and synthetic municipal wastewater samples spiked with 3-CA at 1 and 5 mg L^−1^ (Table 6). The recoveries ranged from 74 to 85% with relative standard deviations (RSD) of 2.38–5.48% (n = 3), demonstrating high accuracy and precision across matrices. Synthetic wastewater, prepared according to Shrestha et al. [66], presented the highest interference due to elevated organic and ionic contents, yet the results remained analytically reliable. These results align with prior studies on mesoporous silica and nanosorbents for D-SPE [67,68], confirming the reliability of BPA-MSNs as an efficient DSPE sorbent for trace-level of 3-CA in environmental samples.

The D-SPE protocol offers effective clean-up and enrichment for 3-CA and related aromatic amines, making it well-suited as a sample preparation step prior to reversed-phase HPLC with UV or MS detection for enhanced selectivity and multi-residue analysis. The performance metrics (Table 5 and Table 6) align with contemporary nanomaterial-based sorbents [11,24,26,69], supporting its practical utility in routine water quality assessment, particularly when coupled with selective detection in complex matrices.

### 3.6. Comparative Performance and Reusability of BPA-MSNs as Sorbent for 3-CA

The adsorption performance of the synthesized bisphenol A-imprinted mesoporous silica nanoparticles (BPA-MSNs) for 3-CA was benchmarked against the literature-reported sorbents to evaluate their efficacy in aqueous remediation (Table 7). Under optimal conditions (pH 6, 25 °C, initial concentration 20 mg L^−1^), BPA-MSNs exhibited a maximum adsorption capacity (q_m_) of 30.2 mg g^−1^, derived from the Langmuir isotherm fitting. This value significantly surpasses those of unmodified clay minerals, including kaolinite (<0.4 mg g^−1^ at pH 5.0–5.5) and Na-montmorillonite (<0.5 mg g^−1^ at pH 8.8–9.3) [70], as well as raw halloysite (5.5% removal efficiency at pH < 5, 60 °C) and its acid-activated counterpart (21.3%) [71]. While treated coffee waste achieved a higher q_m_ of 45.8 mg g^−1^ (pH 7, 25 °C) [72], its non-selective binding and lack of molecular recognition limit the specificity for 3-CA in complex effluents. In contrast, BPA-MSNs combine high capacity with imprinting-induced selectivity, positioning them as a competitive nanomaterial-based sorbent for targeted aromatic amine removal.

**Figure 11:**
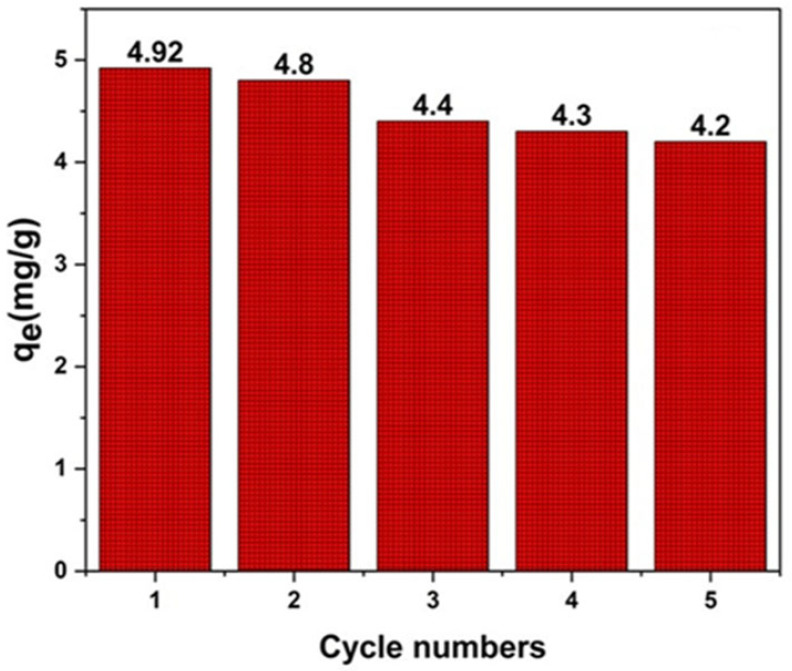
Reusability performance of BPA-MSNs over five consecutive adsorption–desorption cycles for 3-chloroaniline (3-CA). The sorbent retained high adsorption capacity (4.2–4.92 mg g^−1^), demonstrating good stability and regeneration efficiency.

The reusability was investigated over five adsorption–desorption cycles using 100 mg BPA-MSNs, 20 mg L^−1^ 3-CA, 60 min equilibration, and 10 mL ethanol as the desorption solvent (Figure 11). Ethanol was selected due to its superior desorption efficiency (>95% in preliminary tests, Figure 11). The adsorption capacity decreased progressively from 4.92 mg g^−1^ in the first cycle to 4.20 mg g^−1^ in the fifth, retaining >85% of initial performance. This decline likely stems from the partial blockage of imprinted sites, minor surface restructuring, or limited ligand detachment during regeneration. Structural characterization post-cycling indicated minimal loss in the BET surface area (<8%), supporting sustained mesoporous accessibility. These regeneration characteristics are consistent with the ethanol-based recycling of functionalized mesoporous silica reported elsewhere [74,75].

## 4. Conclusions

This study demonstrated the synthesis of BPA-MSN composites with enhanced adsorption affinity toward 3-CA. Structural and spectroscopic analyses verified the efficient MSNs synthesis and the grafting of BPA molecules onto the MSN surface while maintaining the ordered mesoporous framework, thereby ensuring high surface accessibility and dispersibility. The adsorption behavior of 3-CA onto this sorbent followed the Langmuir and Jovanovic isotherm models, and the kinetic evaluation indicated a pseudo-first-order physisorption-dominated mechanism, supported by π–π stacking and hydrogen-bonding interactions. The developed D-SPE protocol showed high analytical performance, achieving low detection limits, satisfactory recovery, and good precision across various water matrices. Thus, it meets the requirements for the monitoring of trace-level of aromatic amines. Additionally, the BPA-MSNs retained more than 90% efficiency after multiple regeneration cycles, confirming their operational stability, cost-effectiveness, and suitability for repeated use. By combining green synthesis with targeted surface engineering, this work introduces a low-cost reusable nanosorbent that bridges laboratory-scale extraction efficiency with practical environmental applicability. BPA-MSNs demonstrate strong potential not only as an advanced DSPE sorbent for sample preparation in environmental monitoring but also as a potentially scalable material platform for the removal of hazardous aromatic amines in wastewater treatment systems.

## Figures and Tables

**Table 1:** Elemental composition of MSNs and BPA-MSNs determined by EDX analysis. The substantial increase in carbon and the appearance of nitrogen in BPA-MSNs confirm successful BPA functionalization of the silica surface.

Sample	Percentage of Weight (%)			
	Si	O	C	N
MSNs	32.1	52.81	15.12	0.0
BPA-MSNs	8.15	27.29	60.31	4.25

**Table 2:** BET surface area, BJH pore volume, most probable pore diameter, and average particle size of the synthesized MSNs and BPA-MSNs.

Sample	BET Surface Area (m^2^/g)	Volume of Pores (cm^3^/g)	Most Probable Pore Diameter (nm)	Particle Size (nm)
MSNs	503	1.9	2.6	17
BPA-MSNs	334	1.4	3.6	15

**Table 3:** Isothermal adsorption parameters describing the equilibrium uptake of 3-chloroaniline (3-CA) onto BPA-MSNs. Adsorption behavior was evaluated using Langmuir, Freundlich, Temkin, Sips, Jossens, and Jovanovic models. Reported values include fitted parameters, determination coefficients (R^2^), and root-mean-square errors (RMSE).

Isotherm Model	Parameters			R^2^	RMSE
**Langmuir** $q_{e}=\frac{q_{max}K_{L}C_{e}}{1+K_{L}C_{e}}$	$q_{max}$ (mg g^−1^)	$K_{L}$ (L mg^−1^)			
	30.26	0.0127		0.98	0.95
**Freundlich** $q_{e}=K_{F}C_{e}^{1/n}$	$K_{F}$ (mg g^−1^ (mg L^−1^)^(1/n)^)	n			
	2.52	2.1		0.96	1.09
**Temkin** $q_{e}=Bln(K_{T}C_{e})$, $B=RT/b_{T}$	$b_{T}$ (J mol^−1^)	$K_{T}$ (L mg^−1^)			
	0.31	5.1		0.93	1.7
**Sips** $q_{e}=\frac{q_{max}(K_{si}C_{e}{)}^{n_{si}}}{1+(K_{si}C_{e}{)}^{n_{si}}}$	$q_{max}$ (mg g^−1^)	$K_{si}$ (L mg^−1^)	$n_{si}$		
	26.58	0.0158	0.8	0.97	0.92
**Jossens** $q_{e}=\frac{K_{j}C_{e}^{n_{j}}}{1+JC_{e}^{n_{j}}}$	$K_{j}$ (L g^−1^)	J (L mg^−1^)	$n_{j}$		
	0.86	0.067	0.88	0.98	0.85
**Jovanovic** $q_{e}=q_{max}(1-e^{-K_{jo}C_{e}})$	$q_{max}$ (mg g^−1^)	$K_{jo}$ (L mg^−1^)			
	23.44	0.014		0.99	0.72

Table notes: $q_{e}$: equilibrium adsorption capacity; $C_{e}$: equilibrium concentration; $q_{max}$: monolayer capacity; $K_{L}$, $K_{F}$, $K_{T}$, $K_{si}$, $K_{j}$, $K_{jo}$: adsorption equilibrium/affinity constants; n, $n_{si}$, $n_{j}$: heterogeneity/intensity factors; $b_{T}$: Temkin heat constant; J: Jossens interaction parameter. All concentrations are in mg L^−1^ and capacities in mg g^−1^; constants are expressed in their respective units as shown in the table.

**Table 4:** Kinetic parameters describing the time-dependent adsorption of 3-chloroaniline (3-CA) onto BPA-MSNs. Adsorption kinetics were fitted using pseudo-first-order, pseudo-second-order, Elovich, and intraparticle diffusion models. Fitted rate constants, model coefficients (R^2^), and RMSE values are presented.

Kinetic Model	Parameters		R^2^	RMSE
**Pseudo-first-order** $\;\;q_{t}=q_{max}(1-e^{-k_{1}t})$	$q_{max}$ (mg g^−1^)	$k_{1}$ (min^−1^)	4.55	0.39
	4.55	0.39	0.99	0.022
**Pseudo-second-order** $\;\;q_{t}=\frac{k_{2}q_{max}t}{1+k_{2}q_{max}t}$	$q_{max}$ (mg g^−1^)	$k_{2}$ (g mg^−1^ min^−1^)	4.65	0.29
	4.65	0.29	0.88	0.08
**Elovich model** $\;\;q_{t}=\frac{1}{b}ln(1+\alpha bt)$	α (mg g^−1^ min^−1^)	b (g mg^−1^)	1.38 × 10^9^	6.03
	1.38E9	6.03	0.68	0.17
**Intraparticle diffusion** $\;\;q_{t}=k_{id}t^{1/2}+C$	$k_{id}$ (mg g^−1^ min^0.5^)	C	0.027	4.24
	0.027	4.24	0.3	0.19

Table notes: $q_{max}$: equilibrium adsorption capacity; $k_{1}$, $k_{2}$: PFO/PSO rate constants; α, b: Elovich initial sorption rate and desorption parameter; $k_{id}$: intraparticle diffusion coefficient; C: boundary-layer thickness. Units follow those listed in the table; $q_{t}$ and $q_{max}$ in mg g^−1^, time in minutes.

**Table 5:** Analytical figures of merit for BPA-MSNs–DSPE method for the of extraction 3-CA.

Parameter	Value
Linear range	0.05–15.0 mg/L
Calibration equation	A = 0.0593 C − 0.000273
R^2^	0.999
LOD (mg/L)	0.016
LOQ (mg/L)	0.05
Preconcentration factor (PF)	20 (50 mL sample, 2.5 mL eluent)
Actual PF	9.54
Recovery (%)	73–85
Precision (RSD, %)	2.38–5.26

**Table 6:** Determination of 3-CA in different water matrices at two spiked levels (*n* = 3) in terms of recovery (%) and precision (RSD in %).

Sample	Added (mg L^−1^)	Found (mg L^−1^)	Recovery (%)	RSD (%)
Bottled water	1	0.85 ± 0.03	85	3.53
	5	4.20 ± 0.10	84	2.38
Ground water	1	0.82 ± 0.03	82	3.67
	5	4.00 ± 0.125	80	3.13
Tap water	1	0.76 ± 0.04	76	5.26
	5	3.72 ± 0.135	74	3.65
Synthetic municipal wastewater *	1	1.1 ± 0.06	110	5.45
	5	5.65 ± 0.31	113	5.48

* Composition based on Shrestha et al. [66].

**Table 7:** Comparative maximum adsorption capacities (q_m_) and removal efficiencies for 3-chloroaniline (3-CA) on BPA-MSNs and literature-reported sorbents under optimized conditions, including reusability performance.

Adsorbent	q_m_ (mg g^−1^), or Efficiency (%)	Conditions (pH, T)	Reusability (Cycles, % Retention)	Reference
Kaolinite	<0.4	5.0–5.5	Not reported	[70]
Na-montmorillonite	<0.5	8.8–9.3	Not reported	[70]
Halloysites	(5.5%)	<5, (60 °C)	Not reported	[71]
Acid-activated halloysite	(21.3%)	(<5, 60 °C)	Not reported	[71]
Coffee waste	45.77	7 (25 °C)	Not regenerable	[72]
Fresh potato peel	0.14	3–9 (30 °C)	Not reported	[73]
BPA-MSNs	30.2	6 (25 °C)	Five cycles, >92%	This work

## Data Availability

Data are available upon request from corresponding author, the data are not publicly available due to privacy or ethical restrictions.

## Abbreviations

The following abbreviations are used in this manuscript:

**Abbreviation**	**Definition**
3-CA	3-Chloroaniline
AADs	Aromatic amine derivatives
BET	Brunauer–Emmett–Teller
BJH	Barrett–Joyner–Halenda
BPA	Bisphenol A
BPA-MSNs	Bisphenol A–functionalized mesoporous silica nanoparticles
CTAC	Cetyltrimethylammonium chloride
D-SPE	Dispersive solid-phase extraction
EDS/EDX	Energy-dispersive X-ray spectroscopy
FESEM	Field-emission scanning electron microscopy
FTIR	Fourier transform infrared spectroscopy
LOD	Limit of detection
LOQ	Limit of quantification
MSNs	Mesoporous silica nanoparticles
PEG	Polyethylene glycol
qmax	Maximum adsorption capacity
SDA	Structure-directing agent
SEM	Scanning electron microscopy
TGA	Thermogravimetric analysis
UV–Vis	Ultraviolet–visible spectroscopy
XRD	X-ray diffraction

## References

[1] Freeman H.S. (2013). Aromatic Amines: Use in Azo Dye Chemistry. Front. Biosci..

[2] Lichtfouse E., Schwarzbauer J., Robert D. (2015). Pollutants in Buildings, Water and Living Organisms.

[3] Özge E., Krupčíková S., Goellner A., Vrana B., Melis M., Melymuk L. (2024). Tracking Aromatic Amines from Sources to Surface Waters. Environ. Sci. Technol. Lett..

[4] Pujar N.K., Premakshi H.G., Shruti L., Pattar S.V., Manisha M., Kamanavalli C.M. (2018). Biodegradation of Chlorpropham and Its Major Products by *Bacillus licheniformis* NKC-1. World J. Microbiol. Biotechnol..

[5] Ferraz E.R.A., de Oliveira G.A.R., de Oliveira D.P. (2012). The Impact of Aromatic Amines on the Environment: Risks and Damages. Front. Biosci. (Elite Ed.).

[6] IARC (1993). Occupational Exposures of Hairdressers and Barbers and Personal Use of Hair Colourants; Some Hair Dyes, Cosmetic Colourants, Industrial Dyestuffs and Aromatic Amines.

[7] Rebelo D., Antunes S.C., Rodrigues S. (2023). The Silent Threat: Exploring the Ecological and Ecotoxicological Impacts of Chlorinated Aniline Derivatives and Their Metabolites on the Aquatic Ecosystem. J. Xenobiot..

[8] Corcia A.D., Samperi R. (1990). Determination of Chloroaniline Traces in Environmental Waters by Selective Extraction with Two Traps in Tandem and Liquid Chromatography. Anal. Chem..

[9] Zheng K., Pan B., Zhang Q., Han Y., Zhang W., Pan B., Xu Z., Zhang Q., Du W., Zhang Q. (2007). Enhanced Removal of *p*-Chloroaniline from Aqueous Solution by a Carboxylated Polymeric Sorbent. J. Hazard. Mater..

[10] Zheng J., Xu S., Wu Z., Wang Z. (2019). Removal of *p*-Chloroaniline from Polluted Waters Using a Cathodic Electrochemical Ceramic Membrane Reactor. Sep. Purif. Technol..

[11] Noormohammadi F., Faraji M., Pourmohammad M. (2022). Determination of Aromatic Amines in Environmental Water Samples by Deep Eutectic Solvent-Based Dispersive Liquid–Liquid Microextraction Followed by HPLC-UV. Arab. J. Chem..

[12] Krupčíková S., Marek S., Kalousková P., Jakub U., Zdeněk Š., Melymuk L., Melis M., Vrana B. (2024). Investigation of Occurrence of Aromatic Amines in Municipal Wastewaters Using Passive Sampling. Sci. Total Environ..

[13] Gosetti F., Chiuminatto U., Zampieri D., Mazzucco E., Marengo E., Gennaro M.C. (2010). A New On-Line Solid Phase Extraction High Performance Liquid Chromatography Tandem Mass Spectrometry Method to Study the Sun Light Photodegradation of Mono-Chloroanilines in River Water. J. Chromatogr. A.

[14] Müller L., Fattore E., Benfenati E. (1997). Determination of Aromatic Amines by Solid-Phase Microextraction and Gas Chromatography–Mass Spectrometry in Water Samples. J. Chromatogr. A.

[15] Li Y., Wei G., Hu J., Liu X., Zhao X., Wang X. (2008). Dispersive Liquid–Liquid Microextraction Followed by Reversed Phase-High Performance Liquid Chromatography for the Determination of Polybrominated Diphenyl Ethers at Trace Levels in Landfill Leachate and Environmental Water Samples. Anal. Chim. Acta.

[16] Jalilian N., Ebrahimzadeh H., Asgharinezhad A.A. (2017). Dispersive Micro-Solid Phase Extraction of Aromatic Amines Based on an Efficient Sorbent Made from Poly(1,8-Diaminonaphthalene) and Magnetic Multiwalled Carbon Nanotubes Composite. J. Chromatogr. A.

[17] AlSuhaimi A.O. (2025). Sustainable Solid-Phase Extractant Based on Spent Coffee Waste-Derived Activated Carbon Functionalized with 1,10-Phenanthroline-5-Amine for Trace Metals from Groundwater Samples. Sustainability.

[18] Omer O.S., Hussein B.H.M., Hussein M.A., Mgaidi A. (2017). Mixture of Illite-Kaolinite for Efficient Water Purification: Removal of As(III) from Aqueous Solutions. Desalin. Water Treat..

[19] Omer O.S., Hussein B.H.M., Ouf A.M., Hussein M.A., Mgaidi A. (2018). An Organified Mixture of Illite-Kaolinite for the Removal of Congo Red from Wastewater. J. Taibah Univ. Sci..

[20] Manousi N., Rosenberg E., Deliyanni E., Zachariadis G.A., Samanidou V. (2020). Magnetic Solid-Phase Extraction of Organic Compounds Based on Graphene Oxide Nanocomposites. Molecules.

[21] González N., Aguinaga V., Domini C.E., Acebal C.C. (2023). Current Trends in Sample Preparation for the Determination of Primary Aromatic Amines in Environmental Samples. Trends Environ. Anal. Chem..

[22] Yankovych H., Vaclavikova M., Melnyk I. (2023). A Review on Adsorbable Organic Halogens Treatment Technologies: Approaches and Application. Sustainability.

[23] Sajid M., Nazal M.K., Ihsanullah I. (2021). Novel materials for dispersive (micro) solid-phase extraction of polycyclic aromatic hydrocarbons in environmental water samples: A review. Anal. Chim. Acta.

[24] Sajid M., Nazal M.K., Adio S.O. (2018). Applications of Nanomaterials in Miniaturized Extraction Techniques. Nanomaterials in Chromatography.

[25] Chisvert A., Cárdenas S., Lucena R. (2019). Dispersive Micro-Solid Phase Extraction. TrAC Trends Anal. Chem..

[26] Ścigalski P., Kosobucki P. (2020). Recent Materials Developed for Dispersive Solid Phase Extraction. Molecules.

[27] Karim A.H., Jalil A.A., Triwahyono S., Sidik S.M., Kamarudin N.H.N., Jusoh R., Jusoh N.W.C., Hameed B.H. (2012). Amino Modified Mesostructured Silica Nanoparticles for Efficient Adsorption of Methylene Blue. J. Colloid Interface Sci..

[28] Alfhaid L.H.K. (2022). Adsorption of Paracetamol in Contaminated Water through pH-Responsive Polymer-Brush-Grafted Mesoporous Silica Nanoparticles. Int. J. Environ. Anal. Chem..

[29] Mahgoub H.A. (2019). Nanoparticles Used for Extraction of Polycyclic Aromatic Hydrocarbons. J. Chem..

[30] Zhang S., Lu F., Ma X., Yue M., Li Y., Liu J., You J. (2018). Quaternary Ammonium-Functionalized MCM-48 Mesoporous Silica as a Sorbent for the Dispersive Solid-Phase Extraction of Endocrine Disrupting Compounds in Water. J. Chromatogr. A.

[31] Narayan R., Nayak U., Raichur A., Garg S. (2018). Mesoporous Silica Nanoparticles: A Comprehensive Review on Synthesis and Recent Advances. Pharmaceutics.

[32] Hwang J., Lee J.H., Chun J. (2021). Facile Approach for the Synthesis of Spherical Mesoporous Silica Nanoparticles from Sodium Silicate. Mater. Lett..

[33] AlMohaimadi K.M., Albishri H.M., Thumayri K.A., AlSuhaimi A.O., Mehdar Y.T.H., Hussein B.H.M. (2024). Facile Hydrothermal Assisted Basic Catalyzed Sol Gel Synthesis for Mesoporous Silica Nanoparticle from Alkali Silicate Solutions Using Dual Structural Templates. Gels.

[34] Matinfar M., Nychka J.A. (2023). A Review of Sodium Silicate Solutions: Structure, Gelation, and Syneresis. Adv. Colloid Interface Sci..

[35] Gorbunova O.V., Baklanova O.N., Gulyaeva T.I., Trenikhin M.V., Drozdov V.A. (2014). Poly(ethylene glycol) as Structure Directing Agent in Sol–Gel Synthesis of Amorphous Silica. Microporous Mesoporous Mater..

[36] Ashour M.M., Mabrouk M., Soliman I.E., Beherei H.H., Tohamy K.M. (2021). Mesoporous Silica Nanoparticles Prepared by Different Methods for Biomedical Applications: Comparative Study. IET Nanobiotechnol..

[37] Yu Q., Hui J., Wang P., Xu B., Zhuang J., Wang X. (2012). Hydrothermal Synthesis of Mesoporous Silica Spheres: Effect of the Cooling Process. Nanoscale.

[38] AlMohaimadi K.M., Albishri H.M., Althumayri K., AlSuhaimi A.O., Hussein B.H.M. (2025). Preparation of Phenanthroline-2-Carbaldehyde Functionalized Mesoporous Silica Nanoparticles as Nanochelator for Solid Phase Extraction of Trace Metals from Wastewater. Arab. J. Chem..

[39] Zhao D., Feng J., Huo Q., Melosh N., Fredrickson G.H., Chmelka B.F., Stucky G.D. (1998). Triblock Copolymer Syntheses of Mesoporous Silica with Periodic 50 to 300 Angstrom Pores. Science.

[40] Setyawan H., Yuwana M., Balgis R. (2015). PEG-Templated Mesoporous Silicas Using Silicate Precursor and Their Applications in Desiccant Dehumidification Cooling Systems. Microporous Mesoporous Mater..

[41] Han Y., Zhang L., Yang W. (2024). Synthesis of Mesoporous Silica Using the Sol–Gel Approach: Adjusting Architecture and Composition for Novel Applications. Nanomaterials.

[42] Joselevich M., Williams F.J. (2008). Synthesis and Characterization of Diazonium Functionalized Nanoparticles for Deposition on Metal Surfaces. Langmuir.

[43] Agho O.B., Okele A.I., Adams D.A., Obadahun J., Enyeribe C. (2016). Application of Bisphenol as a Coupler in the Synthesis of Azo Dyes and Its Assessments on Vegetable Tanned Leather. Anal. Chem. Indian J..

[44] Nuti S., Fernández-Lodeiro A., Galhano J., Oliveira E., Duarte M.P., Capelo-Martínez J.L., Fernández-Lodeiro J. (2024). Tailoring Mesoporous Silica-Coated Silver Nanoparticles and Polyurethane-Doped Films for Enhanced Antimicrobial Applications. Nanomaterials.

[45] Shawky S., Aboalhassan A., Lill H., Bald D., El-Khamisy S., Ebeid E.-Z. (2016). Efficient Loading and Encapsulation of Anti-Tuberculosis Drugs using Multifunctional Mesoporous Silicate Nanoparticles Running Title: Mesoporous Silicate Nanoparticles as Smart Drug Delivery System. J. Nanosci. Curr. Res..

[46] Ermakova E.N., Sysoev S.V., Tsyrendorzhieva I.P., Rakhlin V.I., Kosinova M.L. (2015). Trimethyl(phenyl)silane—A Precursor for Gas Phase Processes of SiC_x_:H Film Deposition: Synthesis and Characterization. Mod. Electron. Mater..

[47] Radi S., Basbas N., Tighadouini S., Bacquet M. (2014). New Polysiloxane Surfaces Modified with Ortho-, Meta- or Para-Nitrophenyl Receptors for Copper Adsorption. J. Surf. Eng. Mater. Adv. Technol..

[48] Dolete G., Purcăreanu B., Mihaiescu D.E., Ficai D., Oprea O.-C., Bîrcă A.C., Chircov C., Vasile B.Ș., Vasilievici G., Ficai A. (2022). A Comparative Loading and Release Study of Vancomycin from a Green Mesoporous Silica. Molecules.

[49] Gorbunova O.V., Baklanova O.N., Gulyaeva T.I. (2020). Porous structure of PEG-mediated silica controlled by solution pH. Microporous Mesoporous Mater..

[50] Amiri A., Saadati-Moshtaghin H.R., Zonoz F.M., Targhoo A. (2017). Preparation and Characterization of Magnetic Wells-Dawson Heteropoly Acid Nanoparticles for Magnetic Solid-Phase Extraction of Aromatic Amines in Water Samples. J. Chromatogr. A.

[51] Liu Z., Mu Q., Sun Y., Gao P., Yu Y., Gao J., Fei Z. (2020). Effective Adsorption of Chloroanilines from Aqueous Solution by *m*-Phenylenediamine Modified Hyper-Cross-Linked Resin: Kinetic, Equilibrium, and Thermodynamic Studies. Colloids Surf. A Physicochem. Eng. Asp..

[52] Langmuir I. (1916). The Constitution and Fundamental Properties of Solids and Liquids. Part I. Solids. J. Am. Chem. Soc..

[53] Freundlich H. (1907). Über die Adsorption in Lösungen. Z. Phys. Chem..

[54] Temkin M.J., Pyzhev V. (1940). Kinetics of the Ammonia Synthesis on Promoted Iron Catalysts. Acta Physicochim. URSS.

[55] Sips R. (1948). On the Structure of a Catalyst Surface. J. Chem. Phys..

[56] Jossens L., Prausnitz J.M., Fritz W., Schlünder E.U., Myers A.L. (1978). Thermodynamics of multi-solute adsorption from dilute aqueous solutions. Chem. Eng. Sci..

[57] Jovanović D.S. (1969). Physical Adsorption of Gases: II. Practical Application of Derived Isotherms for Monolayer and Multilayer Adsorption. Kolloid-Z. Z. Polym..

[58] Rudziński W., Wojciechowski B.W. (1977). On the Jovanović Model of Adsorption: II. The Role of Surface Heterogeneity. Colloid Polym. Sci..

[59] Quiñones I., Guiochon G. (1996). Derivation and Application of a Jovanović–Freundlich Isotherm Model for Single-Component Adsorption on Heterogeneous Surfaces. J. Colloid Interface Sci..

[60] Fathy M., Selim H., Shahawy A.E. (2020). Chitosan/MCM-48 Nanocomposite as a Potential Adsorbent for Removing Phenol from Aqueous Solution. RSC Adv..

[61] Lagergren S. (1898). Zur theorie der sogenannten adsorption geloester stoffe, Kungliga Svenska Vetenskapsakademiens. Handlingar.

[62] Ho K.Y., McKay G., Yeung K.L. (2003). Selective Adsorbents from Ordered Mesoporous Silica. Langmuir..

[63] Juang R.S., Chen M.L. (1997). Application of the Elovich Equation to the Kinetics of Metal Sorption with Solvent-Impregnated Resins. Ind. Eng. Chem. Res..

[64] Weber W.J., Morris J.C. (1963). Kinetics of adsorption on carbon from solution. J. Sanit. Eng. Div..

[65] Al-Ghamdi K., AlSuhaimi A.O., AlMuhaimadi K.M., Mehdar Y.T., Alharbi S.K., Almalky M.A., Hussein B.H. (2025). Applications of Lactobacillus bulgaricus (Lactic Acid Bacteria) as dispersive solid phase sorbent for sample preparation of heavy metals from aqueous samples. Green Anal. Chem..

[66] Shrestha B., Hernandez R., Fortela D.L.B., Sharp W., Chistoserdov A., Gang D., Zappi M.E. (2023). Formulation of a Simulated Wastewater Influent Composition for Use in the Research of Technologies for Managing Wastewaters Generated during Manned Long-Term Space Exploration and Other Similar Situations—Literature-Based Composition Development. BioTech.

[67] Cashin V.B., Eldridge D.S., Yu A., Zhao D. (2018). Surface Functionalization and Manipulation of Mesoporous Silica Adsorbents for Improved Removal of Pollutants: A Review. Environ. Sci. Water Res. Technol..

[68] Santoyo Treviño M.J., Zarazúa S., Płotka-Wasylka J. (2022). Nanosorbents as Materials for Extraction Processes of Environmental Contaminants and Others. Molecules.

[69] Khezeli T., Daneshfar A. (2017). Development of dispersive micro-solid phase extraction based on micro and nano sorbents. TrACTrend Anal. Chem..

[70] Angioi S., Polati S., Roz M., Rinaudo C., Gianotti V., Gennaro M.C. (2005). Sorption Studies of Chloroanilines on Kaolinite and Montmorillonite. Environ. Pollut..

[71] Szczepanik B., Słomkiewicz P., Garnuszek M., Rogala P., Banaś D., Kubala-Kukuś A., Stabrawa I. (2017). Effect of Temperature on Halloysite Acid Treatment for Efficient Chloroaniline Removal from Aqueous Solutions. Clays Clay Miner..

[72] Alsehli B.R. (2023). Removal of 3-Chloroaniline from Aqueous Solution by Treated Coffee Waste: Isotherm Modelling and Thermodynamic Studies. Int. J. Environ. Anal. Chem..

[73] Mohammed N.S., Flowers T.H., Duncan H.J. (2016). Adsorption of 3-Chloroaniline on Potato Skin in Aqueous Solution. Indian J. Mater. Sci..

[74] Islam M.A., Awual M.R., Angove M.J. (2019). A Review on Nickel (II) Adsorption in Single and Binary Component Systems and Future Path. J. Environ. Chem. Eng..

[75] ALOthman Z.A. (2012). A Review: Fundamental Aspects of Silicate Mesoporous Materials. Materials.

